# Fenoxycarb exposure disrupted the reproductive success of the amphipod *Gammarus fossarum* with limited effects on the lipid profile

**DOI:** 10.1371/journal.pone.0196461

**Published:** 2018-04-27

**Authors:** Hélène Arambourou, Inmaculada Fuertes, Emmanuelle Vulliet, Gaëlle Daniele, Patrice Noury, Nicolas Delorme, Khedidja Abbaci, Carlos Barata

**Affiliations:** 1 Irstea Lyon, Riverly Research Unit, Villeurbanne, France; 2 Department of Environmental Chemistry, Institute of Environmental Assessment and Water Research (IDAEA), Spanish Research Council (CSIC), Barcelona, Spain; 3 Univ Lyon, CNRS, Université Claude Bernard Lyon 1, ENS de Lyon, Institut des Sciences Analytiques, UMR 5280, Villeurbanne, France; VIT University, INDIA

## Abstract

Insect growth regulator insecticides mimic the action of hormones on the growth and development of insect pests. However, they can affect the development of non-target arthropods. In the present study, we tested the effects of the growth regulator insecticide fenoxycarb on several endpoints in the freshwater crustacean *Gammarus fossarum* (Amphipoda). Females carrying embryos in their open brood pouch were exposed to 50 μg L^-1^ fenoxycarb throughout the entire oogenesis (i.e. 21 days). After exposure, newborn individuals from exposed embryos were removed from the maternal open brood pouch for lipidomic analysis, while males were added to assess the reproductive success. After fertilization, the lipid profile, energy reserve content (lipids, proteins and glycogen), and activity of phenoloxidase − an enzyme involved in the immune response − were measured in females. No significant effect of fenoxycarb exposure was observed on the lipid profile of both newborn individuals and females, while reproductive success was severely impaired in exposed females. Particularly, precopulatory behavior was significantly reduced and fertilized eggs were unviable. This study highlighted the deleterious effects of the insect growth regulator fenoxycarb on gammarid reproduction, which could have severe repercussions on population dynamics.

## Introduction

The use of growth regulator insecticides having juvenoid activity is increasing because they have a selective mode of action and some pests have developed resistance against classical neurotoxic insecticides [1]. They are used in agriculture for pest management [2], in public health for vector control [3] and in veterinary treatment to prevent pet infestation [4]. Juvenoid insecticides mimic the action of juvenoid hormones on the growth and development of insect pests. Two hormonally regulated pathways are generally targeted: the juvenile hormone pathway and the ecdysteroid hormone pathway [5]. In insects, the juvenile hormone regulates both metamorphosis and reproduction. Therefore, by acting on these hormonally regulated functions, juvenoid insecticides jeopardize successful molting, metamorphosing and reproduction. Nevertheless, juvenoid insecticides can affect the development of non-target arthropods, such as crustaceans. Indeed, crustaceans have a juvenile hormone methyl farnesoate that has a similar chemical structure and may play a role in development similar to the juvenile hormone in insects [6]. In line with this, lipids profile perturbation [7], morphological abnormalities [8], reproduction impairment [9] and reduced vitellogenin genes expression [10,11] were reported in the cladoceran *Daphnia magna* exposed to juvenoid insecticides. In addition, the juvenoid insecticide fenoxycarb was responsible for both growth and lipid content impairment in the mud crab *Rhithropanopeus harrisii* [12] and for reduced fertility in the hymenoptera *Aphytis melinus* [13]. Moreover, tissue alterations and delayed hatching were observed in newborn individuals of the amphipod *Gammarus fossarum* from embryos directly exposed to fenoxycarb [14]. The juvenile hormone is also suspected of playing a key role on the arthropod immune system. In accordance with this, an alteration of the activity of phenoloxidase–an enzyme involved in melanization and arthropod immunity–was reported in the insects *Apis mellifera* [15] and *Rhodnius prolixus* [16] exposed to juvenoid insecticides.

In the present study, we tested the effects of the insecticide fenoxycarb, an analog of the insect juvenile hormone, on two life stages (embryo and adult) of the amphipod *G*. *fossarum*. *Gammarus* sp. are widely distributed in freshwater systems and play a major role in food webs: they not only contribute to the degradation of organic matter, but they also serve as food for many macroinvertebrates, fishes and amphibians [17].

**Fig 1:**
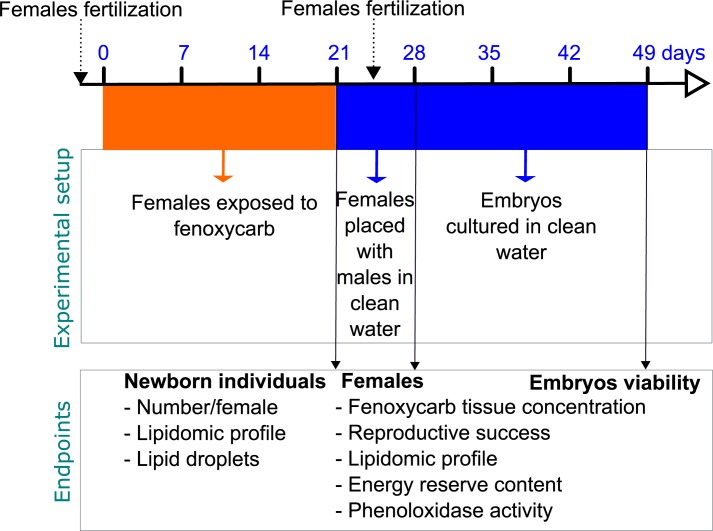
Experimental design and measured endpoints in *G*. *fossarum* exposed to 50 μg L^-1^ fenoxycarb.

In gammarids, both oocyte development and embryo development are synchronized [18]. Females of *G*. *fossarum* carrying embryos in their ventral brood pouch were exposed to 50 μg L^-1^ fenoxycarb throughout the entire oogenesis/embryogenesis cycles (Fig 1). In our previous study, a fenoxycarb concentration of 50 μg L^-1^ was shown to disturb the embryogenesis of *G*. *fossarum* [14]. The study concentration is high but could be detected in water bodies surrounding agricultural areas [19]_._ In the present study, given the potential effect of fenoxycarb on hormonally regulated functions, three endpoints related to reproductive success were evaluated in females previously exposed to fenoxycarb: i) precopulatory behavior, ii) success of fertilization and iii) post-fertilization embryo viability. Furthermore, the effects of fenoxycarb exposure on the lipid profiles of both newborn individuals from exposed embryos and freshly fertilized females were measured. Indeed, given that lipid metabolism is hormonally regulated and that lipids can be mobilized for detoxification processes under toxic exposure, we hypothesize that the lipid profile will be disturbed by the fenoxycarb exposure. Finally, as juvenoid insecticides can interfere with arthropod immunity, phenoloxidase activity was also measured in exposed females.

## Materials and methods

### Collection and maintenance of *G*. *fossarum*

Specimens of *G*. *fossarum* were collected (using a hand net) from a source population used in previous studies in our laboratory [16], which inhabits La Bourbre River at Saint-Clair-de-la-Tour (45°30’N, 5°30’E, France). As this species is not considered as protected in France, specific collection permission for specimens was not required. Organisms with a size ranging from 2.0 mm and 2.5 mm were sorted by sieving and quickly transported to the laboratory in plastic vessels, where they were kept at 14°C with a 16:8-h light:dark photoperiod in 30-L tanks, continuously filled with groundwater and aerated. After 10 days of rearing, precopula pairs were placed in an aquarium containing synthetic water at 14°C. Individuals were monitored daily until the males had fertilized and released the females. Synthetic water used in the present study was the ADaM water [20], composed of: 1.84 mM (CaCl_2_.H_2_O), 0.66 mM NaHCO_3_, 0.001 mM SeO_2_ and 0.333 g.L^-1^ sea salts (Sigma Aldrich) (electrical conductivity = 1000 μS.cm^-1^). Animals were fed *ad libitum* on alder leaves (*Alnus glutinosa*). Alder leaves were collected in nearby Lyon (France), dried at room temperature, and soaked in groundwater for two weeks before use.

### Experimental set up

A stock solution of fenoxycarb was prepared by adding fenoxycarb (pestanal, Sigma-Aldrich) to acetone (for HPLC, Carbo-Elba). Then, the fenoxycarb concentration of 50 μg L^-1^ was made by diluting the stock solution in synthetic water. Acetone concentration (0.005%, v/v) was kept constant in the fenoxycarb and control conditions. Previous experiments using the same gammarid population showed no significant effect of acetone at this concentration of exposure [18, 21]. Two days after fertilization, females carrying embryos in their open brood pouch were placed individually in 125 mL glass jars filled with 100 mL of the test medium (either the solvent control or a 50 μg L^-1^ fenoxycarb solution, n_control_ = 45, n_fenoxycarb_ = 58). Females carrying embryos were exposed for 21 days at 14±1°C with a 16:8 h light:dark photoperiod. Half of the medium was renewed every 3 days. Animals were fed *ad libitum* on alder leaves. At 14°C, embryos hatched in the brood pouch 23 days after fertilization [14]. Hatched embryos rest inside the maternal brood pouch before being released. To ensure that they had not eaten before the lipid analysis, newborns were gently removed from the brood pouch 23 days after female fertilization (Fig 1) by sweeping the blunted end of a Pasteur pipette through the pouch in the posterior-to-anterior direction.

Once newborns have been removed, each female was placed individually in a 125 mL glass jar filled with 100 mL of synthetic water, and one mature male (i.e. males which were in precopula in the gammarid culture) was added to promote fertilization. Animals were fed *ad libitum* on alder leaves and 50% of the medium was renewed every 3 days. Three endpoints related to reproductive success were assessed: i) precopulatory behavior, ii) success of fertilization and iii) post-fertilization embryo viability. Precopulatory behavior refers to male clasp and swimming with the female until female molting. This behavior ensures successful sexual contact during the post ecdysis period when mating can occur [22]. Each couple was monitored daily during the reproductive period (i.e. for 7 days) to ensure that the males had fertilized and released the females. Fertilization was considered successful when fertilized eggs were released into the female brood pouch. Each day, newly fertilized females were placed in tanks containing synthetic water and embryos were gently removed from the maternal brood pouch following two days of fertilization by sweeping the blunted end of a Pasteur pipette through the pouch [23]. Fifty newborn individuals were then placed in 25 ceramic well-plates in saline synthetic water at 14°C for culturing (Fig 1), as described in Arambourou et al. [14]. Females were frozen at -80°C until further analysis. Half of the embryo medium was renewed every 3 days and embryos were considered viable when they successfully broke their chorion.

### Fenoxycarb analyses

Fenoxycarb concentrations in the water phase were checked at the beginning of the exposure and after 3 days of exposure, just before the media was renewed. Fenoxycarb analysis was performed by liquid chromatograph-mass spectrometer (LC-MS/MS) on an H-Class UPLC system (Waters) coupled to a Xevo TQ-S triple quadrupole mass spectrometer (Waters). The chromatographic column was a Kinetex 1.7 μm EVO C18 100A (Phenomenex). The mobile phase (A) was composed of 0.4 mM ammonium acetate + 0.01% acetic acid in water and the mobile phase (B) was methanol. The injection volume was set at 2 μL. MS/MS detection was performed in multiple reaction monitoring (MRM) mode with the electrospray ionization source operating in the positive mode (electrospray ionization, ESI+). The MRM transitions were 302 → 116 (quantification transition) and 302 → 88 (confirmation transition). The quantification limit for fenoxycarb was 0.04 μg L^-1^. At the beginning of exposure, measured concentrations were <0.04 μg L^-1^ and 61 μg L^-1^ for the control and the fenoxycarb exposed groups, respectively. After 3 days of exposure, measured concentrations were <0.04 μg L^-1^ and 41 μg L^-1^ for the control and the fenoxycarb exposed groups, respectively.

After reproductive success observations, fenoxycarb concentration was also measured in females. For that purpose, three pooled females were ground and homogenized. Then, a QuEChERS extraction, combining an initial liquid-liquid extraction in acetonitrile, water, heptane and a citrate buffering salt, was carried out. This was followed by a purification step of the acetonitrile phase on a PSA/C18 dispersive solid extraction phase. Fenoxycarb was quantified with LC-MS/MS using a matrix-matched calibration, under the same conditions as those described for fenoxycarb measurement in the water phase. Three replicates per condition were analyzed.

### Energy reserve content and phenoloxidase activity in females

Frozen females were analyzed for lipid content, protein content and phenoloxidase activity by spectrometry as described in Arambourou and Stoks [24]. Glycogen content was measured by spectrometry according to the method reported by Gómez-Lechon et al. [25].

### Lipidomic analysis in both newborns and females

Lipidomic analysis was performed as described by Jordão et al. [26] with minor modifications. Three replicates, consisting of a pool of 40 newborn individuals, per condition were analyzed. For females, three replicates of two pooled individuals per condition were considered. Briefly, each replicate was homogenized in 1 mL of chloroform:methanol (2:1) with 2,6-di-*tert*-butyl-4-methylphenol (BHT; 0.01%) as an antioxidant. Lipid extraction was performed using a modification of Folch’s method [27]. Briefly, 100 μL of the homogenized sample was mixed with 750 μL of chloroform and 250 μL of methanol. To semi-quantify the lipids, internal standards (S1 Table) were also added. Samples were then dried under N_2_. Lipid extracts were solubilized in 300 μL methanol. The LC-MS/MS consisted of a Waters Aquity UPLC system connected to a LCT premier orthogonal accelerated time-of-flight mass spectrometer (Waters) operated in positive and negative ESI mode. Full-scan spectra from 50 to 1,800 Da were obtained. Mass accuracy and reproducibility were maintained using an independent reference spray (LockSpray; Waters). A 100-mm × 2.1-mm i.d., 1.7-μm C8 Acquity UPLC BEH (Waters) analytical column was used. In newborn individuals, a total of 129 lipids, distributed as follows, were identified and semi-quantified: 70 triacylglycerols (TAG), 10 diacylglycerols (DAG), 34 phosphatidylcholines (PC), 3 lysophosphatidylcholines (LPC), 5 phosphatidylethanolamines (PEA), 3 sphingomyelins(SM) and 3 phosphatidylserine (PS). In females, a total of 122 lipids distributed as follows, were identified and semi-quantified: 62 TAGs, 10 DAGs, 34 PCs, 4 LPCs, 5 PEAs, 3 SMs and 4 PSs.

### Mass and lipid droplets in newborns

Newborn gammarids freshly extracted from the female brood pouch were gently dried on a paper, pooled in groups of five individuals, and weighed using an ultra-microbalance (Sartorius Ultra Micro Balance MSU2.7S000DM, readability 0.1 μg, repeatability ± 0.25 μg).

Lipid droplets in newborn individuals were visualized using a stock solution of Nile red staining (technical grade, Sigma-Aldrich) prepared with 10 mg of Nile red to 100 mL of acetone (for HPLC, Carbo-Elba). Just before use, the working solution was prepared by diluting the stock solution to 2 mg L^-1^ in synthetic water. Live individuals were then exposed to the Nile red working solution in the dark for 3 h at 14°C. Stained individuals were immobilized between the microscope slide and the cover slide, and images of the entire animal were taken at 5 × magnification for visualization of lipid droplets. Fluorescence images were obtained using a Leica DM 2500 with a L5 filter cuber (EX 480/40, EM 527/30) [28]. Twenty individuals per condition were analyzed.

### Statistical treatment

All statistical analyses were performed using R software [29]. Differences in juvenile production, mass, lipid droplets, energy reserve content and phenoloxidase activity between the control group and the fenoxycarb-exposed group were assessed using a non-parametric Mann-Whitney-Wilcoxon test, which is a robust test requiring minimal assumptions [30]. For testing differences of mortality and viability a Chi-squared proportion test was used. Lipidome composition (lipid concentration expressed as pmol/mg of fresh tissue) was visualized with a principal component analysis (PCA) using the FactoMineR package and the difference between groups was assessed with an ANOVA on the principal component scores, followed by a post-hoc Tuckey test.

## Results

### Life-history traits

After 21 days of exposure, the adult female survival rate in the fenoxycarb-exposed group (90%) was not significantly different from the control (89%) (Proportion test, χ^2^ = 8.10^−31^, p = 1; Table 1). A fenoxycarb concentration of 1.9 ng g^-1^ of fresh tissue was detected in the exposed females (Table 1). The bioconcentration factor—which is the concentration in the tissues relative to the concentration in the surrounding medium (water)—was 0.03 L kg^-1^. No significant effects of fenoxycarb exposure on either female mass (Mann-Whitney-Wilcoxon test, W = 69, p = 0.95; Table 1) or newborns production (Mann-Whitney-Wilcoxon test, W = 130, p = 0.36; Table 1) were found.

**Table 1:** Life-history traits in *G*. *fossarum* exposed to 50 μg L^-1^ fenoxycarb. Mean±standard deviation. In parentheses, number of individuals or pooled sample analyzed.

	Female survival rate (%)	Tissue concentrations (ng/g fresh tissue)	Female mass (mg)	Number of newborns/female	Newborns mass (μg)
Control	89	<0.15 (n = 3)	13.5±3.9 (n = 14)	17±5 (n = 12)	138.4±13.8 (n = 32)
Fenoxycarb	90	1.90±0.14 (n = 3)	14.4±3.3 (n = 10)	15±5 (n = 18)	141.3±11.7 (n = 32)

### Reproductive success and embryos viability

**Fig 2:**
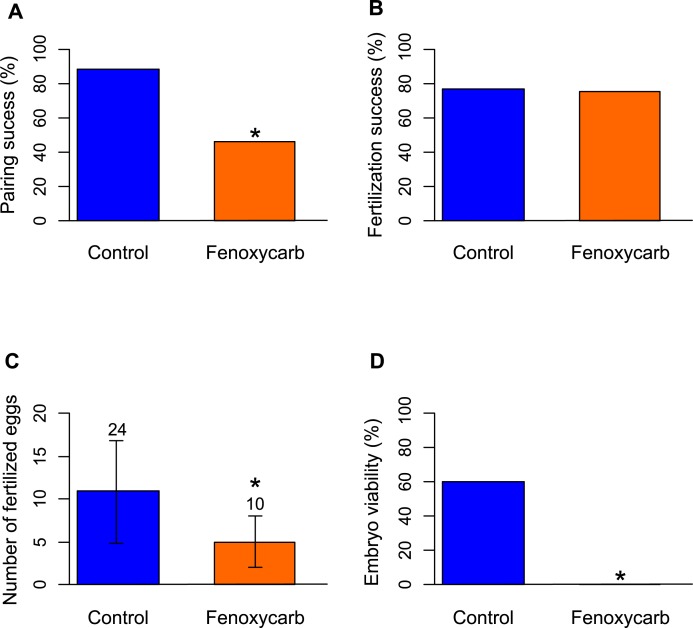
Reproductive parameters in the control group and in the 50 μg L^-1^ fenoxycarb-exposed group. Pairing success in percent (A), success of fertilization in paired gammarid in percent (B), number of fertilized eggs per female (mean±standard deviation; numbers above the bars indicate the number of females that were observed) (C) and embryo viability in percent (50 embryos/condition) (D). Asterisks indicate significant differences compared to the control group.

Females previously exposed to fenoxycarb and maintained in control water with males for reproduction, exhibited an altered precopulatory behavior. Indeed, within 7 days after introducing the males, 88% of the gammarids were in precopula in the control group while only 46% of the gammarids were in precopula in the fenoxycarb-exposed group (Proportion test, χ^2^ = 15, p<0.001; Fig 2A). Moreover, average number of embryos produced was reduced from 11 in the control group to six in the fenoxycarb-exposed group (Mann-Whitney-Wilcoxon test, W = 206, p = 0.001; Fig 2C). Survival of embryos cultured in well-plates was 60% in the control group, which is of the same order of magnitude as our previous observations [14]. Embryos produced by females exposed during oogenesis were not viable: all were dead after 21 days of rearing (proportion test, χ^2^ = 40, p<0.001, Fig 2D).

### Energy reserve content and phenoloxidase activity in females

**Fig 3:**
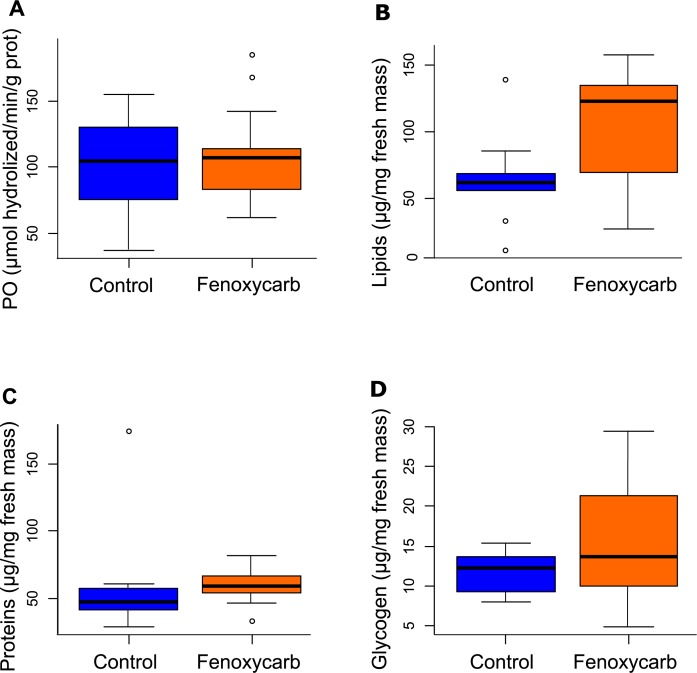
Biochemical markers in control group and in the 50 μg L^-1^ fenoxycarb-exposed group. Phenoloxidase activity (A), lipid content (B), protein content (C) and glycogen content (D). Ten females and 15 females were analyzed in the control and fenoxycarb-exposed groups, respectively.

No significant differences between the control and the fenoxycarb-exposed group for either phenoloxidase activity (Mann-Whitney-Wilcoxon test, W = 74, p = 0.98; Fig 3A) or energy reserve content (Mann-Whitney-Wilcoxon tests, lipids: W = 41, p = 0.06; proteins: W = 40, p = 0.06 and glycogen: W = 54, p = 0.25; Fig 3B, 3C and 3D) were found.

### Lipidomic analysis in both newborns and females

**Fig 4:**
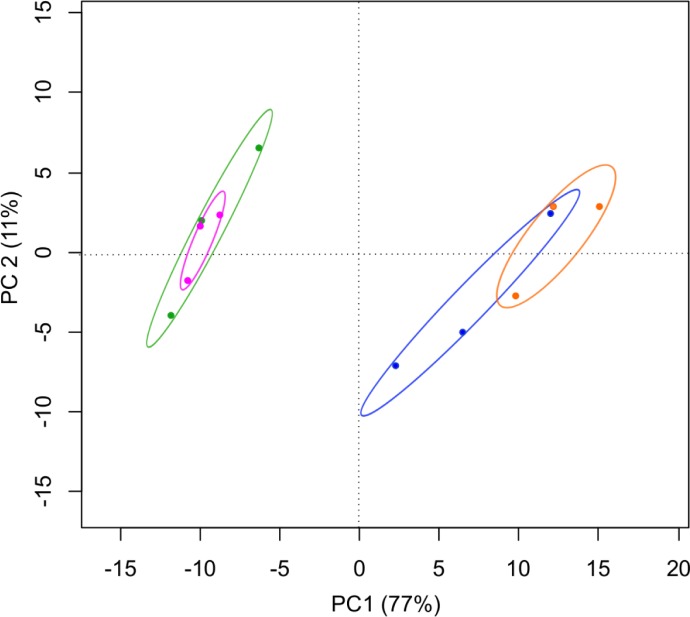
PCA scores of the lipid profiles plotted on the two first components of the PCA. Females in the control (green) and fenoxycarb-exposed group (pink) and newborns in the control (blue) and fenoxycarb-exposed group (orange).

Abundant lipids in both females and newborns included TAGs (energy reserve) and PCs (component of biological membrane) (S2 Table). The two first axes of the PCA explained 88% of the total variance (Fig 4). The first axis is positively correlated with TAG compounds and negatively correlated with DAG compounds, while the second axis is positively correlated with PC compounds. They were differences in lipidome composition between newborn individuals and the females (ANOVA on the PC1 coordinates, F_3,8_ = 39, p<0.01; Fig 4). Newborns exhibited a higher content of TAGs and a lower content of DAGs than females. No significant differences in lipid profile were observed between control and fenoxycarb-exposed groups for either newborn individuals or females (post hoc Tukey tests, both p>0.05).

### Mass and lipid droplets in newborns

**Fig 5:**
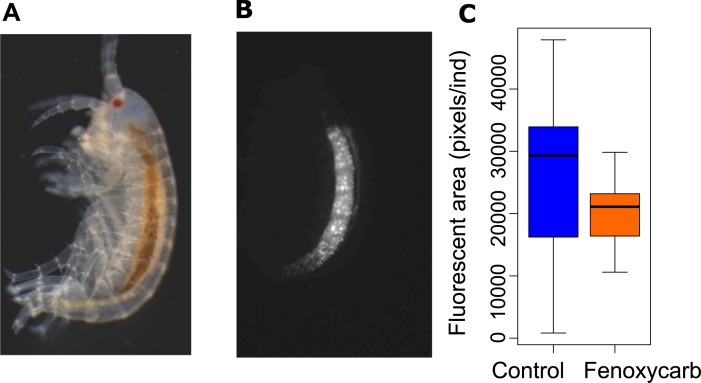
Observation of lipid droplets in newborns. Newborn individual observed under a stereomicroscope (A), newborn individual observed with a fluorescence microscope after Nile red staining (lipid droplets are concentrated into the hepatopancreas) (B) and fluorescent area in the two study conditions (20 individuals/condition) (C).

No significant difference in newborns mass was observed between the control and the fenoxycarb-exposed group (Mann-Whitney-Wilcoxon test, W = 446, p = 0.38; Table 1). Nile red staining revealed an accumulation of lipid droplets in the hepatopancreas (Fig 5B). No significant difference in lipid content was observed between the control and the fenoxycarb-exposed group (Mann-Whitney-Wilcoxon test, W = 267, p = 0.07; Fig 5C).

## Discussion

The reproductive success of *G*. *fossarum* was strongly affected by exposure to 50 μg L^-1^ of fenoxycarb, while both exposed newborn individuals and females did not exhibit any significant alteration of their lipid profiles.

There was little accumulation of fenoxycarb in female tissue. Yet, given the low solubility of fenoxycarb in water (log Kow = 4.07), this compound is expected to be accumulated in tissues rich in lipids. This result suggested metabolization/excretion of fenoxycarb, which is not surprising because fenoxycarb is suspected of mimicking the action of the crustacean hormone methyl farnesoate, and thus might be metabolized by methyl farnesoate esterase.

Interference of fenoxycarb with hormonally regulated pathways might be responsible for the impairment of precopulatory behavior in exposed females. In amphipods, precopulatory behavior has been shown to be severely altered by insecticide exposure [31,32]. Borowsky and Borowsky [33] showed that the most important stimulus for male reproductive behavior in *Gammarus palustris* is the female exoskeleton, suggesting contact pheromones in this species may exist. Evidence for pheromonal control of mating behavior is, however, conflicting in gammarids. For example, Lyes [34] noted that pair formation in *Gammarus duebeni* was regulated by a pheromone produced by the female, while Hartnoll and Smith [22] were unable to demonstrate sex pheromone activity in *G*. *duebeni*. More recently, Cornet et al. [35] stated that the female molt stage, which varies in levels of ecdysone and vitellogenin, influences pairing decisions. Therefore, reduced ability of gammarids to enter a precopulatory state following female exposure to fenoxycarb might result from either an alteration of pheromone production or a perturbation of the exoskeleton status related to impairment of ecdysone and vitellogenin metabolic pathways. A previous study using specimens of the same gammarid population showed a significant reduction of vitellogenic-like proteins in females exposed to juvenile hormone methyl farnesoate [36]. Similarly, an impairment of vitellogenin genes expression was reported in the cladorecan *D*. *magna* exposed to fenoxycarb [10,11].

Juvenoids have also been shown to disrupt lipid storage—which is a hormonally regulated function—in the crustaceans *D*. *magna* [37] and *R*. *harrisii* [12]. In the present study, no significant difference in the lipid profile was observed between females from the control group and females from the fenoxycarb-exposed group. Females were analyzed just after fertilization, which means after having transferred the vitellus to the freshly fertilized eggs. If fenoxycarb mainly affects vitellogenesis processes as stated above, it is likely that the lipid profile was merely altered in the fertilized eggs. Therefore, further studies could perform a lipidomic analysis in freshly fertilized eggs. Given that in gammarids the vitellus is the main energetic pathway that fuels embryo development [38], the disruption of vitellogenesis in females exposed to fenoxycarb might also explain the lack of viable embryos produced. We did not detect any significant alteration of the phenoloxidase activity in females previously exposed to fenoxycarb. In the literature, phenoloxidase activity impairment was reported in insects exposed to juvenoid insecticides [15,16] but for exposure concentrations much higher than used in the present study. In addition, given that females were placed with males in water without fenoxycarb to assess the reproductive success for a 7-day period, phenoloxidase might have recovered from the induced toxic effect. Further experiments are needed to test the direct effect of fenoxycarb on phenoloxidase activity.

As reported for females, the lipid profile of newborn individuals from exposed embryos was not significantly altered by the fenoxycarb exposure. Hamdoun and Epel [39] suggested the embryo stage might not be the most sensitive stage within the life cycle of an organism, as is often claimed. The embryo stage has been shown to be less sensitive to toxic exposure than the adult stage in the cladoceran *D*. *magna* exposed to heavy metals [40] and in the isopod *Asellus aquaticus* exposed to cadmium [41]. Embryos that are directly in contact with the surrounding medium, such as in the amphipod *G*. *fossarum*, appear resistant to environmental stress by pathogens, UV radiation and xenobiotic chemicals (39). *G*. *fossarum* embryos are protected by an outer layer, the chorion, which might limit fenoxycarb uptake, as has been observed in embryos of the brine shrimp *Artemia* exposed to chlorpyrifos [42]. In the fenoxycarb-exposed dormant eggs of *D*. *magna*, despite the outer layer, fenoxycarb was taken up to a concentrations of 13 ng/egg [8]. Nonetheless, as suggested by the authors, measured toxicant levels may be influenced by chemicals adsorbed on the outer layer.

## Conclusions

Reproductive success, especially reproductive behavior, was severely impaired in females of *G*. *fossarum* exposed to fenoxycarb. Reproductive behavior disruption might be caused by an interference of fenoxycarb with hormonally regulated pathways, which could also reduce embryo viability although further experiments are needed to test this hypothesis. Overall, these results underlined the substantial sensitivity of the oogenesis period in *G*. *fossarum*. By dramatically reducing reproductive success, juvenoid exposure could have severe repercussions on the population dynamics of gammarids, and consequently on ecosystem functioning.

## Supporting information

S1 Table Internal standards added to the sample to semi quantify lipid compounds. (DOCX)

S2 Table Semi quantification of lipid compounds (pmol/mg of fresh mass) in newborn individuals and in females. Three replicates of 40 animals of each condition (control and fenoxycarb) were analyzed for newborn individuals and three replicates of two animals of each condition (control and fenoxycarb) were analyzed for females. TAG: triacylglycerols, DAG: diacylglycerols, PC: phosphatidylcholines, LPC: lysophosphatidylcholines, PEA: phosphatidylethanolamines, SM: sphingomyelins, and PS: phosphatidylserine.
(DOCX)
