# Supplementary material for: Fenoxycarb exposure disrupted the reproductive success of the amphipod Gammarus fossarum with limited effects on the lipid profile
Source: PLoS One. 2018 Apr 27;13(4):e0196461. doi: 10.1371/journal.pone.0196461 (PMC5922543; doi:10.1371/journal.pone.0196461)
Supplement: S1 Table — (DOCX) [file pone.0196461.s001.docx]

**Supporting information**

**S1 Table. Internal standards added to the sample to semi quantify lipid compounds.**

| Internal standard | pmol added |
| --- | --- |
| 1,2,3-17:0 TAG | 200 |
| 1,3-17:0 D5 DAG | 166 |
| 16:0 D31-18:1 PC | 126 |
| 17:0 Lyso PC | 120 |
| 16:0 D31-18:1 PEA | 133 |
| 16:0 D31-18:1 PS | 123 |
