# Supplementary material for: Fenoxycarb exposure disrupted the reproductive success of the amphipod Gammarus fossarum with limited effects on the lipid profile
Source: PLoS One. 2018 Apr 27;13(4):e0196461. doi: 10.1371/journal.pone.0196461 (PMC5922543; doi:10.1371/journal.pone.0196461)
Supplement: S2 Table — Three replicates of 40 animals of each condition (control and fenoxycarb) were analyzed for newborn individuals and three replicates of two animals of each condition (control and fenoxycarb) were analyzed for females. TAG: triacylglycerols, DAG: diacylglycerols, PC: phosphatidylcholines, LPC: lysophosphatidylcholines, PEA: phosphatidylethanolamines, SM: sphingomyelins, and PS: phosphatidylserine. (DOCX) [file pone.0196461.s002.docx]

**Supporting information**

**S2 Table. Semi quantification of lipid compounds (pmol/mg of fresh mass) in newborn individuals and in females.** Three replicates of 40 animals of each condition (control and fenoxycarb) were analyzed for newborn individuals and three replicates of two animals of each condition (control and fenoxycarb) were analyzed for females. TAG: triacylglycerols, DAG: diacylglycerols, PC: phosphatidylcholines, LPC: lysophosphatidylcholines, PEA: phosphatidylethanolamines, SM: sphingomyelins, and PS: phosphatidylserine.

|  | | | Newborn individuals | | | | | | Females | | | | | |
| --- | --- | --- | --- | --- | --- | --- | --- | --- | --- | --- | --- | --- | --- | --- |
|  |  |  | Control | | | Fenoxycarb | | | Control | | | Fenoxycarb | | |
| Abbreviation | | Exact mass | Replicate 1 | Replicate 2 | Replicate 3 | Replicate 1 | Replicate 2 | Replicate 3 | Replicate 1 | Replicate 2 | Replicate 3 | Replicate 1 | Replicate 2 | Replicate 3 |
| TAG | 40:0 | 694.61 | 10.6 | 7.7 | 7.4 | 11.5 | 8.8 | 9.5 | 3.6 | 2.4 | 7.6 | 3.3 | 2.3 | 5.0 |
| TAG | 42:0 | 722.64 | 11.2 | 11.4 | 10.3 | 12.1 | 13.0 | 13.1 | 6.6 | 4.0 | 15.7 | 6.0 | 3.8 | 10.3 |
| TAG | 42:1 | 720.63 | 8.3 | 7.2 | 5.7 | 8.9 | 8.2 | 7.3 | 2.5 | 1.3 | 11.4 | 2.2 | 1.2 | 7.5 |
| TAG | 44:0 | 750.67 | 24.6 | 25.0 | 22.9 | 26.6 | 28.5 | 29.3 | 18.8 | 12.6 | 40.3 | 17.1 | 11.9 | 26.6 |
| TAG | 44:1 | 748.66 | 55.5 | 55.8 | 42.3 | 60.1 | 63.8 | 54.3 | 12.9 | 7.4 | 44.3 | 11.7 | 7.0 | 29.2 |
| TAG | 44:2 | 746.64 | 24.6 | 25.7 | 19.1 | 26.7 | 29.4 | 24.5 | 3.9 | 2.1 | 19.8 | 3.5 | 2.0 | 13.1 |
| TAG | 46:0 | 778.71 | 38.3 | 37.0 | 31.4 | 41.5 | 42.3 | 40.2 | 35.7 | 27.5 | 49.3 | 32.5 | 26.0 | 32.5 |
| TAG | 46:1 | 776.69 | 147.6 | 118.1 | 77.1 | 159.8 | 134.9 | 98.7 | 48.0 | 28.3 | 128.8 | 43.7 | 26.7 | 85.0 |
| TAG | 46:2 | 774.67 | 112.2 | 116.3 | 111.2 | 121.4 | 132.9 | 142.5 | 25.5 | 15.2 | 69.2 | 23.2 | 14.3 | 45.6 |
| TAG | 46:3 | 772.66 | 56.5 | 57.0 | 41.6 | 61.2 | 65.2 | 53.2 | 5.0 | 3.4 | 24.0 | 4.5 | 3.2 | 15.9 |
| TAG | 46:4 | 770.64 | 22.5 | 22.5 | 15.8 | 24.4 | 25.7 | 20.3 | 0.0 | 0.0 | 0.0 | 0.0 | 0.0 | 0.0 |
| TAG | 46:5 | 768.63 | 8.6 | 9.2 | 5.9 | 9.3 | 10.5 | 7.5 | 0.0 | 0.0 | 0.0 | 0.0 | 0.0 | 0.0 |
| TAG | 48:0 | 806.74 | 56.8 | 60.7 | 48.3 | 61.5 | 69.4 | 61.9 | 37.2 | 35.0 | 45.6 | 33.9 | 33.1 | 30.1 |
| TAG | 48:1 | 804.72 | 243.8 | 263.5 | 197.9 | 264.0 | 301.0 | 253.5 | 52.9 | 56.4 | 259.0 | 48.1 | 53.3 | 170.9 |
| TAG | 48:2 | 802.71 | 308.4 | 256.9 | 209.2 | 333.9 | 293.5 | 268.0 | 59.1 | 48.8 | 232.0 | 53.8 | 46.1 | 153.1 |
| TAG | 48:3 | 800.69 | 167.3 | 158.3 | 154.3 | 181.1 | 180.9 | 197.8 | 24.6 | 12.8 | 73.2 | 22.4 | 12.1 | 48.3 |
| TAG | 48:4 | 798.67 | 174.7 | 160.7 | 128.2 | 189.2 | 183.6 | 164.2 | 10.3 | 6.0 | 46.3 | 9.3 | 5.7 | 30.5 |
| TAG | 48:5 | 796.66 | 67.8 | 80.3 | 53.3 | 73.4 | 91.7 | 68.2 | 2.0 | 0.4 | 11.8 | 1.9 | 0.3 | 7.8 |
| TAG | 48:6 | 794.64 | 19.9 | 23.0 | 13.8 | 21.6 | 26.2 | 17.7 | 0.0 | 0.0 | 0.0 | 0.0 | 0.0 | 0.0 |
| TAG | 50:0 | 834.77 | 103.8 | 137.4 | 77.6 | 112.4 | 157.0 | 99.4 | 32.6 | 32.9 | 105.9 | 29.7 | 31.1 | 69.9 |
| TAG | 50:1 | 832.75 | 604.0 | 802.4 | 591.6 | 653.9 | 916.7 | 758.0 | 104.4 | 69.0 | 410.3 | 95.0 | 65.2 | 270.8 |
| TAG | 50:2 | 830.74 | 1240.0 | 1473.4 | 1171.8 | 1342.5 | 1683.2 | 1501.3 | 147.1 | 89.4 | 570.0 | 133.8 | 84.4 | 376.2 |
| TAG | 50:3 | 828.72 | 780.2 | 812.0 | 712.0 | 844.7 | 927.7 | 912.3 | 62.7 | 67.7 | 232.5 | 57.0 | 64.0 | 153.5 |
| TAG | 50:4 | 826.71 | 407.8 | 433.1 | 300.1 | 441.5 | 494.8 | 384.6 | 39.3 | 25.1 | 81.5 | 35.8 | 23.7 | 53.8 |
| TAG | 50:5 | 824.69 | 320.1 | 275.6 | 249.3 | 346.6 | 314.9 | 319.5 | 11.9 | 5.3 | 44.3 | 10.9 | 5.0 | 29.2 |
| TAG | 50:6 | 822.67 | 212.8 | 198.2 | 153.2 | 230.4 | 226.5 | 196.3 | 4.0 | 1.9 | 15.2 | 3.7 | 1.8 | 10.0 |
| TAG | 50:7 | 820.66 | 57.4 | 63.2 | 43.1 | 62.1 | 72.1 | 55.2 | 0.0 | 0.0 | 0.0 | 0.0 | 0.0 | 0.0 |
| TAG | 52:0 | 862.80 | 65.0 | 93.6 | 66.4 | 70.4 | 106.9 | 85.1 | 0.0 | 0.0 | 0.0 | 0.0 | 0.0 | 0.0 |
| TAG | 52:1 | 860.78 | 368.8 | 455.7 | 349.2 | 399.2 | 520.7 | 447.5 | 72.2 | 53.9 | 177.7 | 65.7 | 50.9 | 117.3 |
| TAG | 52:2 | 858.77 | 1970.3 | 2322.3 | 1935.0 | 2133.2 | 2653.1 | 2479.2 | 293.1 | 199.5 | 766.7 | 266.7 | 188.4 | 506.0 |
| TAG | 52:3 | 856.75 | 1966.2 | 2200.2 | 2061.0 | 2128.7 | 2513.6 | 2640.7 | 198.9 | 163.1 | 590.6 | 181.0 | 154.1 | 389.8 |
| TAG | 52:4 | 854.74 | 1151.3 | 1342.4 | 1214.8 | 1246.5 | 1533.7 | 1556.5 | 114.2 | 100.3 | 239.0 | 103.9 | 94.7 | 157.8 |
| TAG | 52:5 | 852.72 | 695.3 | 826.8 | 677.2 | 752.8 | 944.6 | 867.6 | 60.6 | 63.7 | 105.8 | 55.2 | 60.2 | 69.8 |
| TAG | 52:6 | 850.71 | 371.7 | 505.8 | 305.9 | 402.4 | 577.9 | 391.9 | 19.9 | 16.5 | 42.7 | 18.1 | 15.6 | 28.2 |
| TAG | 52:7 | 848.69 | 271.3 | 310.6 | 230.7 | 293.8 | 354.8 | 295.6 | 3.6 | 2.4 | 8.9 | 3.3 | 2.2 | 5.9 |
| TAG | 52:8 | 846.67 | 127.6 | 139.9 | 86.2 | 138.2 | 159.9 | 110.5 | 0.0 | 0.0 | 0.0 | 0.0 | 0.0 | 0.0 |
| TAG | 52:9 | 844.66 | 42.0 | 43.6 | 28.8 | 45.4 | 49.8 | 36.9 | 0.0 | 0.0 | 0.0 | 0.0 | 0.0 | 0.0 |
| TAG | 54:0 | 890.83 | 0.0 | 0.0 | 0.0 | 0.0 | 0.0 | 0.0 | 9.1 | 5.0 | 15.9 | 8.3 | 4.7 | 10.5 |
| TAG | 54:1 | 888.81 | 123.1 | 142.5 | 109.3 | 133.2 | 162.8 | 140.0 | 29.6 | 44.2 | 70.4 | 26.9 | 41.7 | 46.5 |
| TAG | 54:2 | 886.80 | 475.9 | 572.5 | 449.3 | 515.3 | 654.1 | 575.6 | 120.6 | 72.3 | 266.5 | 109.8 | 68.3 | 175.9 |
| TAG | 54:3 | 884.78 | 1178.2 | 1333.1 | 1137.9 | 1275.6 | 1523.0 | 1457.9 | 293.8 | 171.5 | 694.5 | 267.3 | 162.0 | 458.4 |
| TAG | 54:4 | 882.77 | 1162.8 | 1364.5 | 1248.6 | 1258.9 | 1558.9 | 1599.7 | 112.0 | 120.7 | 309.0 | 101.9 | 114.0 | 203.9 |
| TAG | 54:5 | 880.75 | 1158.8 | 1328.8 | 1115.2 | 1254.6 | 1518.1 | 1428.8 | 156.5 | 102.4 | 167.0 | 142.4 | 96.7 | 110.2 |
| TAG | 54:6 | 878.74 | 1326.6 | 1466.1 | 1109.7 | 1436.2 | 1674.9 | 1421.8 | 94.3 | 98.9 | 96.1 | 85.8 | 93.4 | 63.4 |
| TAG | 54:7 | 876.72 | 667.8 | 558.0 | 567.4 | 723.0 | 637.5 | 727.0 | 41.8 | 40.7 | 45.5 | 38.0 | 38.4 | 30.0 |
| TAG | 54:8 | 874.71 | 324.1 | 326.7 | 271.4 | 350.9 | 373.2 | 347.8 | 7.9 | 6.1 | 1.4 | 7.2 | 5.8 | 0.9 |
| TAG | 54:9 | 872.69 | 213.2 | 234.9 | 162.8 | 230.8 | 268.3 | 208.6 | 0.0 | 0.0 | 0.0 | 0.0 | 0.0 | 0.0 |
| TAG | 54:10 | 870.67 | 75.0 | 90.6 | 55.3 | 81.2 | 103.5 | 70.9 | 0.0 | 0.0 | 0.0 | 0.0 | 0.0 | 0.0 |
| TAG | 54:11 | 868.66 | 16.2 | 19.8 | 12.7 | 17.6 | 22.7 | 16.2 | 0.0 | 0.0 | 0.0 | 0.0 | 0.0 | 0.0 |
| TAG | 56:1 | 916.85 | 0.0 | 0.0 | 0.0 | 0.0 | 0.0 | 0.0 | 8.9 | 3.0 | 18.5 | 8.1 | 2.8 | 12.2 |
| TAG | 56:2 | 914.83 | 181.6 | 212.7 | 179.5 | 196.6 | 243.0 | 229.9 | 99.6 | 37.0 | 169.4 | 90.6 | 34.9 | 111.8 |
| TAG | 56:3 | 912.81 | 202.8 | 249.2 | 172.8 | 219.5 | 284.7 | 221.4 | 96.3 | 41.6 | 175.2 | 87.6 | 39.3 | 115.6 |
| TAG | 56:4 | 910.80 | 209.2 | 195.5 | 203.8 | 226.5 | 223.3 | 261.1 | 60.0 | 28.1 | 85.5 | 54.6 | 26.5 | 56.4 |
| TAG | 56:5 | 908.78 | 297.5 | 349.1 | 285.0 | 322.0 | 398.9 | 365.1 | 49.7 | 25.4 | 68.8 | 45.3 | 24.0 | 45.4 |
| TAG | 56:6 | 906.77 | 597.6 | 614.5 | 455.1 | 647.0 | 702.1 | 583.0 | 49.3 | 38.3 | 54.0 | 44.9 | 36.2 | 35.6 |
| TAG | 56:7 | 904.75 | 939.8 | 946.9 | 724.6 | 1017.5 | 1081.8 | 928.4 | 53.8 | 48.4 | 59.1 | 49.0 | 45.7 | 39.0 |
| TAG | 56:8 | 902.74 | 567.7 | 587.1 | 503.2 | 614.6 | 670.7 | 644.7 | 27.4 | 22.1 | 15.8 | 24.9 | 20.9 | 10.4 |
| TAG | 56:9 | 900.72 | 354.9 | 297.8 | 265.8 | 384.2 | 340.2 | 340.6 | 9.1 | 8.8 | 0.1 | 8.2 | 8.3 | 0.1 |
| TAG | 56:10 | 898.71 | 273.4 | 276.6 | 224.9 | 296.0 | 316.0 | 288.1 | 0.0 | 0.0 | 0.0 | 0.0 | 0.0 | 0.0 |
| TAG | 56:11 | 896.69 | 132.3 | 143.3 | 79.5 | 143.2 | 163.7 | 101.8 | 0.0 | 0.0 | 0.0 | 0.0 | 0.0 | 0.0 |
| TAG | 56:12 | 894.67 | 21.9 | 28.3 | 16.0 | 23.7 | 32.3 | 20.5 | 0.0 | 0.0 | 0.0 | 0.0 | 0.0 | 0.0 |
| TAG | 58:3 | 940.85 | 58.2 | 70.3 | 61.4 | 63.0 | 80.4 | 78.7 | 25.0 | 10.7 | 41.7 | 22.8 | 10.1 | 27.5 |
| TAG | 58:4 | 938.83 | 73.7 | 81.8 | 69.6 | 79.8 | 93.5 | 89.1 | 14.1 | 6.7 | 21.7 | 12.8 | 6.3 | 14.3 |
| TAG | 58:5 | 936.81 | 61.1 | 65.4 | 56.2 | 66.1 | 74.7 | 72.1 | 7.4 | 3.8 | 11.4 | 5.5 | 8.1 | 6.6 |
| TAG | 58:6 | 934.80 | 191.9 | 189.6 | 157.8 | 207.7 | 216.6 | 202.2 | 16.0 | 7.4 | 15.9 | 14.6 | 7.0 | 10.5 |
| TAG | 58:7 | 932.78 | 296.8 | 247.6 | 258.0 | 321.4 | 282.9 | 330.5 | 21.9 | 18.6 | 20.2 | 20.0 | 17.6 | 13.3 |
| TAG | 58:8 | 930.77 | 277.5 | 261.5 | 243.3 | 300.4 | 298.7 | 311.8 | 14.2 | 14.8 | 7.5 | 12.9 | 14.0 | 4.9 |
| TAG | 58:9 | 928.75 | 278.2 | 271.4 | 207.2 | 301.2 | 310.0 | 265.5 | 6.5 | 7.4 | 2.4 | 5.9 | 7.0 | 1.6 |
| TAG | 58:10 | 926.74 | 253.3 | 239.7 | 185.1 | 274.2 | 273.8 | 237.1 | 3.1 | 2.9 | 2.4 | 2.8 | 2.7 | 1.6 |
| TAG | 58:11 | 924.72 | 222.8 | 270.8 | 170.9 | 241.2 | 309.4 | 219.0 | 2.4 | 2.5 | 2.5 | 2.2 | 2.4 | 1.7 |
| TAG | 58:12 | 922.71 | 144.1 | 122.8 | 77.7 | 156.0 | 140.2 | 99.6 | 0.0 | 0.0 | 0.0 | 0.0 | 0.0 | 0.0 |
| TAG | 58:13 | 920.69 | 40.8 | 42.8 | 22.7 | 44.2 | 48.9 | 29.1 | 0.0 | 0.0 | 0.0 | 0.0 | 0.0 | 0.0 |
| DAG | 32:0 | 568.51 | 36.0 | 36.6 | 35.0 | 54.5 | 57.1 | 43.1 | 161.8 | 146.2 | 213.2 | 159.7 | 141.9 | 167.1 |
| DAG | 34:0 | 596.54 | 64.7 | 65.2 | 59.1 | 98.0 | 101.8 | 72.8 | 209.7 | 184.6 | 201.8 | 207.1 | 179.1 | 158.1 |
| DAG | 34:1 | 594.52 | 239.7 | 286.8 | 225.0 | 362.8 | 447.7 | 277.1 | 103.3 | 79.2 | 167.8 | 102.0 | 76.9 | 131.5 |
| DAG | 34:2 | 592.51 | 111.1 | 133.5 | 113.4 | 168.1 | 208.3 | 139.7 | 30.7 | 23.2 | 48.5 | 30.4 | 22.5 | 38.0 |
| DAG | 34:3 | 590.49 | 42.9 | 54.9 | 38.7 | 64.9 | 85.6 | 47.6 | 0.0 | 0.0 | 0.0 | 0.0 | 0.0 | 0.0 |
| DAG | 36:0 | 624.57 | 70.3 | 89.1 | 79.9 | 106.4 | 139.2 | 98.4 | 107.1 | 104.8 | 147.6 | 105.7 | 101.7 | 115.7 |
| DAG | 36:1 | 622.55 | 22.7 | 26.3 | 19.9 | 34.4 | 41.1 | 24.5 | 9.5 | 6.2 | 15.6 | 9.4 | 6.0 | 12.2 |
| DAG | 36:2 | 620.54 | 119.2 | 143.1 | 110.1 | 180.4 | 223.4 | 135.6 | 74.1 | 49.5 | 115.3 | 73.1 | 48.0 | 90.3 |
| DAG | 36:3 | 618.52 | 92.5 | 105.5 | 98.0 | 140.0 | 164.6 | 120.7 | 65.6 | 47.3 | 112.6 | 64.8 | 45.9 | 88.3 |
| DAG | 36:5 | 614.49 | 79.6 | 91.9 | 63.5 | 120.4 | 143.4 | 78.2 | 24.2 | 17.9 | 40.9 | 23.9 | 17.3 | 32.1 |
| PC | 30:0 | 705.53 | 25.7 | 44.1 | 18.6 | 28.7 | 42.7 | 39.1 | 21.1 | 13.3 | 32.7 | 20.8 | 17.3 | 28.1 |
| PC | 32:0 | 733.56 | 201.1 | 255.1 | 128.8 | 224.6 | 247.0 | 270.2 | 111.6 | 106.6 | 129.9 | 109.5 | 138.6 | 111.3 |
| PC | 32:1 | 731.55 | 261.0 | 403.1 | 174.1 | 291.5 | 390.3 | 365.2 | 174.9 | 121.3 | 123.9 | 171.7 | 157.8 | 106.2 |
| PC | 32:2 | 729.53 | 34.7 | 55.6 | 22.5 | 38.7 | 53.8 | 47.1 | 25.7 | 19.1 | 40.2 | 25.2 | 24.8 | 34.4 |
| PC | 34:0 | 761.59 | 40.2 | 58.1 | 29.5 | 44.9 | 56.2 | 61.9 | 23.0 | 14.6 | 28.3 | 22.6 | 19.0 | 24.2 |
| PC | 34:1 | 759.58 | 1994.8 | 3415.7 | 1712.0 | 2227.7 | 3307.1 | 3590.4 | 1201.5 | 939.3 | 1488.1 | 1179.2 | 1222.1 | 1274.8 |
| PC | 34:2 | 757.56 | 621.1 | 916.0 | 499.8 | 693.6 | 886.8 | 1048.1 | 409.3 | 299.3 | 531.3 | 401.7 | 389.4 | 455.1 |
| PC | 34:3 | 755.55 | 272.2 | 455.5 | 185.4 | 304.0 | 441.0 | 388.7 | 209.1 | 175.8 | 170.5 | 205.2 | 228.7 | 146.1 |
| PC | 34:4 | 753.53 | 45.2 | 69.0 | 26.8 | 50.4 | 66.8 | 56.1 | 36.0 | 32.1 | 45.0 | 35.4 | 41.8 | 38.6 |
| PC | 34:5 | 751.52 | 19.7 | 33.1 | 10.4 | 22.0 | 32.1 | 21.9 | 8.2 | 5.2 | 14.4 | 8.0 | 6.7 | 12.3 |
| PC | 36:1 | 787.61 | 293.6 | 406.2 | 230.6 | 327.9 | 393.2 | 483.6 | 213.7 | 166.9 | 153.0 | 209.8 | 217.1 | 131.1 |
| PC | 36:2 | 785.59 | 433.5 | 782.9 | 326.9 | 484.2 | 758.0 | 685.6 | 538.8 | 363.4 | 781.7 | 528.8 | 472.7 | 669.6 |
| PC | 36:3 | 783.58 | 431.7 | 759.4 | 404.1 | 482.1 | 735.2 | 847.5 | 413.4 | 341.0 | 535.6 | 405.7 | 443.7 | 458.8 |
| PC | 36:4 | 781.56 | 378.2 | 629.4 | 332.1 | 422.3 | 609.4 | 696.5 | 350.7 | 274.7 | 320.7 | 344.2 | 357.3 | 274.7 |
| PC | 36:5 | 779.55 | 456.5 | 783.3 | 367.2 | 509.9 | 758.4 | 770.2 | 188.9 | 135.8 | 214.0 | 185.4 | 176.7 | 183.3 |
| PC | 36:6 | 777.53 | 145.9 | 237.4 | 100.4 | 163.0 | 229.8 | 210.5 | 70.0 | 48.8 | 85.8 | 68.7 | 63.5 | 73.5 |
| PC | 38:1 | 815.64 | 27.8 | 51.0 | 21.7 | 31.0 | 49.3 | 45.6 | 39.3 | 18.5 | 41.5 | 38.5 | 24.1 | 35.6 |
| PC | 38:2 | 813.62 | 85.6 | 144.2 | 69.0 | 95.6 | 139.6 | 144.8 | 111.2 | 55.2 | 119.8 | 109.2 | 71.8 | 102.7 |
| PC | 38:3 | 811.61 | 86.4 | 145.9 | 69.0 | 96.4 | 141.2 | 144.6 | 123.0 | 72.5 | 119.3 | 120.8 | 94.3 | 102.2 |
| PC | 38:4 | 809.59 | 58.5 | 91.9 | 41.8 | 65.4 | 89.0 | 87.6 | 208.5 | 139.5 | 186.4 | 204.7 | 181.5 | 159.7 |
| PC | 38:5 | 807.58 | 464.3 | 839.2 | 363.2 | 518.6 | 812.5 | 761.7 | 322.9 | 263.9 | 331.7 | 316.9 | 343.4 | 284.1 |
| PC | 38:6 | 805.56 | 928.3 | 1608.9 | 783.1 | 1036.7 | 1557.8 | 1642.2 | 622.9 | 446.4 | 582.4 | 611.4 | 580.7 | 498.9 |
| PC | 38:7 | 803.55 | 278.4 | 462.1 | 274.3 | 310.9 | 447.4 | 575.3 | 183.3 | 129.5 | 194.8 | 179.9 | 168.5 | 166.9 |
| PC | 38:8 | 801.53 | 115.7 | 192.6 | 77.8 | 129.2 | 186.5 | 163.3 | 88.0 | 73.5 | 82.9 | 86.3 | 95.6 | 71.1 |
| PC | 38:9 | 799.52 | 12.5 | 20.2 | 7.7 | 14.0 | 19.6 | 16.1 | 11.3 | 4.7 | 12.3 | 11.1 | 6.1 | 10.5 |
| PC | 40:2 | 841.66 | 35.7 | 54.4 | 27.4 | 39.9 | 52.7 | 57.4 | 41.9 | 21.7 | 39.9 | 41.1 | 28.2 | 34.2 |
| PC | 40:3 | 839.64 | 27.3 | 42.9 | 25.4 | 30.5 | 41.6 | 53.2 | 29.1 | 13.0 | 23.8 | 28.5 | 16.9 | 20.4 |
| PC | 40:4 | 837.63 | 29.3 | 52.2 | 26.6 | 32.7 | 50.5 | 55.7 | 43.8 | 26.5 | 49.9 | 43.0 | 34.5 | 42.8 |
| PC | 40:5 | 835.61 | 54.5 | 82.9 | 39.4 | 60.8 | 80.3 | 82.6 | 109.0 | 75.6 | 125.2 | 107.0 | 98.4 | 107.2 |
| PC | 40:6 | 833.59 | 175.9 | 264.2 | 132.6 | 196.5 | 255.8 | 278.1 | 267.6 | 172.1 | 307.5 | 262.7 | 223.9 | 263.4 |
| PC | 40:7 | 831.58 | 373.1 | 693.5 | 322.7 | 416.6 | 671.5 | 676.9 | 331.7 | 246.6 | 335.2 | 325.5 | 320.8 | 287.1 |
| PC | 40:8 | 829.56 | 231.1 | 390.8 | 185.4 | 258.1 | 378.3 | 388.9 | 194.2 | 143.5 | 186.4 | 190.6 | 186.7 | 159.7 |
| PC | 40:9 | 827.55 | 168.6 | 272.8 | 135.1 | 188.3 | 264.2 | 283.3 | 115.4 | 85.4 | 91.9 | 113.3 | 111.0 | 78.8 |
| PC | 40:10 | 825.53 | 177.2 | 345.3 | 179.6 | 197.8 | 334.3 | 376.6 | 93.6 | 60.8 | 90.3 | 91.8 | 79.1 | 77.3 |
| LPC | 16:0 | 495.33 | 14.6 | 14.4 | 19.7 | 7.6 | 62.9 | 180.1 | 32.4 | 20.5 | 20.6 | 26.5 | 19.1 | 14.7 |
| LPC | 18:1 | 521.35 | 13.1 | 13.6 | 12.8 | 24.6 | 24.5 | 22.4 | 67.5 | 30.8 | 53.9 | 55.2 | 28.7 | 38.4 |
| LPC | 18:2 | 519.33 | 8.9 | 8.3 | 9.2 | 16.7 | 14.9 | 16.0 | 32.0 | 16.1 | 27.1 | 26.2 | 15.0 | 19.3 |
| LPC | 18:3 | 517.32 | 0.0 | 0.0 | 0.0 | 0.0 | 0.0 | 0.0 | 13.7 | 9.5 | 11.7 | 11.3 | 8.8 | 8.3 |
| PEA | 34:1 | 717.53 | 14.1 | 14.6 | 12.5 | 18.0 | 19.0 | 16.4 | 52.5 | 44.5 | 93.2 | 62.6 | 46.7 | 83.9 |
| PEA | 34:2 | 715.52 | 93.4 | 109.1 | 95.8 | 71.9 | 83.5 | 99.2 | 71.3 | 68.0 | 106.3 | 85.1 | 71.4 | 95.7 |
| PEA | 36:2 | 743.55 | 224.1 | 259.4 | 198.1 | 411.4 | 395.0 | 303.9 | 160.5 | 156.2 | 233.3 | 191.4 | 164.0 | 210.0 |
| PEA | 36:3 | 741.53 | 201.7 | 247.3 | 210.6 | 370.2 | 376.6 | 323.1 | 168.1 | 166.8 | 228.9 | 200.5 | 175.1 | 206.1 |
| PEA | 36:4 | 739.52 | 93.0 | 110.9 | 82.9 | 170.7 | 168.9 | 127.2 | 87.1 | 101.9 | 119.2 | 103.9 | 107.0 | 107.3 |
| PEA | 36:5 | 737.50 | 136.1 | 153.5 | 129.3 | 249.9 | 233.7 | 198.4 | 70.7 | 49.7 | 114.0 | 84.3 | 52.2 | 102.6 |
| SM | 14:0 | 674.54 | 26.7 | 37.3 | 18.5 | 29.1 | 36.1 | 38.1 | 19.5 | 15.8 | 21.9 | 19.3 | 20.7 | 18.6 |
| SM | 16:0 | 702.57 | 83.2 | 122.3 | 65.6 | 90.7 | 118.5 | 135.2 | 73.9 | 44.3 | 73.8 | 72.9 | 57.9 | 62.5 |
| SM | 18:0 | 730.60 | 164.9 | 256.5 | 135.0 | 179.6 | 248.4 | 278.1 | 144.0 | 120.7 | 99.4 | 142.0 | 157.7 | 84.3 |
| PS | 34:0 | 763.54 | 134.6 | 128.1 | 127.1 | 135.8 | 106.2 | 143.4 | 54.4 | 68.9 | 53.3 | 68.8 | 61.1 | 59.8 |
| PS | 38:0 | 819.60 | 208.8 | 218.5 | 182.9 | 334.5 | 290.8 | 249.1 | 136.2 | 164.0 | 181.2 | 172.1 | 145.3 | 203.6 |
| PS | 38:1 | 817.58 | 82.9 | 91.9 | 73.0 | 52.8 | 61.9 | 58.9 | 83.1 | 105.8 | 132.4 | 105.1 | 93.7 | 148.7 |
| PS | 40:1 | 845.61 | 0.0 | 0.0 | 0.0 | 0.0 | 0.0 | 0.0 | 36.7 | 42.4 | 58.2 | 46.3 | 37.6 | 65.3 |
